# Functional Linear and Nonlinear Brain–Heart Interplay during Emotional Video Elicitation: A Maximum Information Coefficient Study

**DOI:** 10.3390/e21090892

**Published:** 2019-09-14

**Authors:** Vincenzo Catrambone, Alberto Greco, Enzo Pasquale Scilingo, Gaetano Valenza

**Affiliations:** Department of Information Engineering and Bioengineering and Robotics Research Center “E.Piaggio”, University of Pisa, Largo Lucio Lazzarino, 56126 Pisa, Italy; alberto.greco@centropiaggio.unipi.it (A.G.); e.scilingo@centropiaggio.unipi.it (E.P.S.); g.valenza@ing.unipi.it (G.V.)

**Keywords:** brain–heart interplay, brain–heart axis, heart rate variability, EEG, maximum information coefficient, coupling, nonlinearity, emotion, psychophysiology

## Abstract

Brain and heart continuously interact through anatomical and biochemical connections. Although several brain regions are known to be involved in the autonomic control, the functional brain–heart interplay (BHI) during emotional processing is not fully characterized yet. To this aim, we investigate BHI during emotional elicitation in healthy subjects. The functional linear and nonlinear couplings are quantified using the maximum information coefficient calculated between time-varying electroencephalography (EEG) power spectra within the canonical bands (δ,θ,α,β and γ), and time-varying low-frequency and high-frequency powers from heartbeat dynamics. Experimental data were gathered from 30 healthy volunteers whose emotions were elicited through pleasant and unpleasant high-arousing videos. Results demonstrate that functional BHI increases during videos with respect to a resting state through EEG oscillations not including the γ band (>30 Hz). Functional linear coupling seems associated with a high-arousing positive elicitation, with preferred EEG oscillations in the θ band ([4,8) Hz) especially over the left-temporal and parietal cortices. Differential functional nonlinear coupling between emotional valence seems to mainly occur through EEG oscillations in the δ,θ,α bands and sympathovagal dynamics, as well as through δ,α,β oscillations and parasympathetic activity mainly over the right hemisphere. Functional BHI through δ and α oscillations over the prefrontal region seems primarily nonlinear. This study provides novel insights on synchronous heartbeat and cortical dynamics during emotional video elicitation, also suggesting that a nonlinear analysis is needed to fully characterize functional BHI.

## 1. Introduction

Emotions in humans are fundamental psychophysiological adaptations to the external environment [1,2,3]. According to the James–Lange theory [4], emotions are cognitive reactions to physiological peripheral responses to stimuli, whereas they are peripheral reactions to a central nervous system (CNS) processing to stimuli according to Cannon–Bard and Papez–MacLean theories [5]. Indeed, although contradicting, these theories all assert that a significant interplay between CNS and peripheral systems exists, particularly referring to the autonomous nervous system (ANS) activity on cardiovascular control [3]. The ANS maintains the body homeostasis and thus regulates emotional processes through the continuous activity of its sympathetic and parasympathetic branches [2]. CNS-ANS interplay has recently been scientifically formalized through the definition of the so-called central autonomic network, which comprises CNS areas known to be involved in the autonomic control including brainstem nuclei and a number of forebrain regions [6,7,8].

Autonomic Nervous System activity on cardiovascular regulation, comprising sympathetic and parasympathetic (vagal) dynamics, involves complex cortical, subcortical, and medullary efferents [9,10]. For instance, the insular cortex, which participates in the control of both ANS branches, plays a primary role in emotional processing [11,12]. Indeed, autonomic alterations and cardiovascular instabilities may be caused by insular damage after stroke [13]. Furthermore, medial prefrontal cortex is activated with mental stress, which is known to be associated with cardiac arrhythmias [14,15]. On the other hand, heart afferent inputs strongly affect cerebral functions associated with perception, cognition, or emotions through areas including amygdala, hypothalamus, and thalamus [16,17]. These exemplary BHI involve nonlinear relationships through activity synchronisation between amygdala and cardiac dynamics [18].

CNS studies on emotions have mostly been carried out through functional neuroimaging and electroencephalography (EEG) [19,20,21], whereas ANS studies primarily focused on heart rate variability (HRV) analysis [3,21,22,23]. Studies investigating brain–heart interplay (BHI) have focused on the concurrent quantification of EEG-, and HRV-derived features, also in the frame of an emotional scenario [9,24,25,26,27,28,29,30,31,32,33]. According to the so-called frequency domain paradigm, the EEG signal can be analysed by deriving the power within the following canonical bands δ∈[0,4)Hz,θ∈[4,8)Hz,α∈[8,13)Hz,β∈[13,30)Hz,γ>30Hz, whereas the power in a low-frequency (HRV-LF, 0.04–0.15 Hz) band and a high-frequency (HRV-HF, 0.15–0.40 Hz) band may be calculated from HRV series to derive markers of sympathovagal and parasympathetic dynamics, respectively [23,34].

Recent findings highlight that EEG power in the θ band correlates with HRV-derived features when recorded over the temporal regions [35], which are sensitive to emotions [36,37,38]. EEG power in the α band correlates with HRV complexity indices during relaxation [26], whereas the power in the β band is functionally linked to HRV-LF and HRV-HF powers during yoga [31], emotional imageshow [35], sleep [32], or physical stress [28].

Most of the aforementioned studies have quantified BHI by calculating the correlation between EEG and HRV measures at a group-wise level, thus, neglecting intra-subject coupling and related time-varying brain–heart dynamics. Also, whether the functional nature of functional BHI follows a linear or nonlinear coupling function is not fully understood yet. Note that a nonlinear coupling function describes a functional relationship between variables that are not associated with input–output proportionality. In a recent study [35], we investigated functional linear or nonlinear BHI by computing the maximum information coefficient (MIC) between EEG and HRV power series in healthy subjects whose emotions were elicited through images. MIC, in fact, may be equivalent to the Pearson correlation coefficient in the case of a purely linear coupling while also detecting different kinds of nonlinear couplings [39]. Therefore, a MIC value close to one may be indistinctly associated with a high functional linear or nonlinear coupling between systems, possibly limiting the interpretation of results.

Here, we overcome this limitation by combining the Pearson correlation coefficient with MIC [39], thus quantifying fully linear or nonlinear functional BHI during emotional elicitation. To this end, we use high-arousing emotional video clips as suggested in [40,41,42,43]. Videos, in fact, are known to enhance the emotional experience through a multisensory elicitation (see [44] for review), and BHI during emotional video processing is still unknown to us. Particularly, we study differential BHI occurring between positive and negative videos, and express experimental results as *p*-values topographic maps, as well as continuous Z-score topographic maps to comply with latest recommendations on *p*-value interpretation and thresholding [45,46]. These results, followed by Discussion and Conclusion, and Materials and Methods sections follow below.

## 2. Results

We report experimental results on 30 healthy subjects whose emotions were elicited using high-arousing, pleasant and unpleasant videos (see details in Section 5.1). The following frequency bands are considered for further analysis (all expressed in Hz): LF=[0.04,0.15) and HF=[0.15,0.4] for heartbeat dynamics, and δ=[0.5,4),θ=[4,8),α=[8,13),β=[13,30),γ=[30,60] for EEG signals.

Series of time-varying power spectra were computed throughout the recordings at each frequency band and for each subject. A characterisation of the spectral power of EEG and heartbeat dynamics at different frequency bands is provided in Table 1, in which the reported values refer to a grand average between subjects/channels at each experimental phase.

Functional linear or nonlinear BHI was then quantified by computing the MIC between EEG-derived and HRV-derived power series during the emotional elicitation when normalized (divided) by the relative power estimated during the resting state.

Group-wise (median) MIC values are shown in the Supplementary Information as topographical maps for positive and negative elicitation sessions. At a qualitative level, MIC ranges increase at higher EEG frequencies, with grand average values as high as 0.38 for oscillations in the γ band. Moreover, higher group-wise MIC values seem to occur over the right hemisphere for EEG oscillations in the β and γ bands, whereas central brain areas seems to be more functionally correlated to heartbeat dynamics than others especially in θ, α and β bands.

Statistical testing over MIC values was then considered to quantitatively investigate significant differences between positive and negative videos. To this end, Z-score and corresponding thresholded *p*-value topographic maps were built from non-parametric Wilcoxon tests for paired samples. A *p*-value related to the null hypothesis of equal median between samples was deemed to be significant if lower than 0.05 following a correction for multiple comparison based on permutation tests with 1000 permutations.

Results indicate that MIC values increase during emotional videos with respect to a resting state for EEG oscillations below 30 Hz (see Supplementary Information for details).

**Figure 1:**
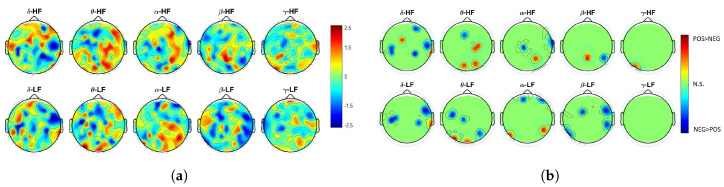
(**a**) Z-score topographic maps from the Wilcoxon non-parametric test for paired data applied to MIC estimates between positive and negative elicitations. (**b**) Associated significant *p*-values. Green areas indicate non-significant (corrected) *p*-values (p>0.05), whereas yellow/red (blue) areas indicate that BHI was significantly higher during positive (negative) valence than during negative (positive) one.

Results also indicate that differences in functional linear or nonlinear coupling between positive and negative videos are in the temporal, parietal, and pre-frontal regions (see Figure 1a,b). Particularly, the left-temporal lobe shows significant differences considering the EEG-θ and HRV-LF coupling, as well as in the EEG-δ coupling with HRV-LF and HRV-HF powers. The right-parietal cortex seems to be involved in the differential functional coupling with EEG-δ, EEG-α, and EEG-β oscillations, whereas the pre-frontal lobes seem to be involved in the differential coupling between EEG-δ and HRV-HF power, and EEG-α and HRV-LF power. Functional linear or nonlinear BHI through cortical oscillations in the γ band did not show significant differences between positive and negative elicitations.

**Figure 2:**
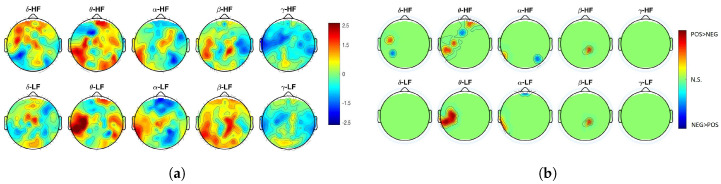
(**a**) Z-score topographic maps from the Wilcoxon non-parametric test for paired data applied to Pearson linear correlation coefficient ρ estimates between positive and negative elicitations. (**b**) Associated significant *p*-values. Green areas indicate non-significant (corrected) *p*-values (p>0.05), whereas yellow/red (blue) areas indicate that functional linear BHI was significantly higher during positive (negative) valence than during negative (positive) one.

To further investigate which cortical region is mainly associated with a linear functional coupling compared to a nonlinear interplay, we performed a BHI analysis based on the Pearson linear correlation coefficient ρ. Results from this analysis, which followed the same statistical procedure applied to MIC, are shown in Figure 2a,b.

Major differences in functional linear coupling between negative and positive valence are with EEG oscillations in the θ band, especially over the left-temporal and parietal cortices. A higher functional linear coupling is associated with a high-arousing positive video.

**Figure 3:**
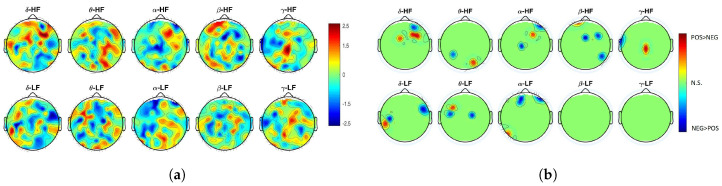
(**a**) Z-score topographic maps from the Wilcoxon non-parametric test for paired data applied to MIC$-\rho^{2}$ nonlinear estimates between positive and negative elicitations. (**b**) Associated significant *p*-values. Green areas indicate non-significant (corrected) *p*-values (p>0.05), whereas yellow/red (blue) areas indicate that functional nonlinear BHI was significantly higher during positive (negative) valence than during negative (positive) one.

For a comprehensive characterization of the BHI, we also investigated functional nonlinear coupling between EEG and HRV powers by calculating the nonlinear index $MIC-\rho^{2}$. Results for this nonlinear index expressed as Z-score and corresponding thresholded *p*-value topographic maps are shown in Figure 3a,b, respectively.

Significant changes in functional nonlinear BHI between positive and negative videos are mainly linked to EEG oscillations below 30 Hz (i.e., δ,θ,α and β band). In the δ band, significant changes are mainly over the temporal lobes considering a functional BHI with the HRV-LF band. The EEG-θ leads the BHI over the dorso-parietal lobes through the HRV-HF power, as well as over the ventro-parietal lobes through the HRV-LF power. Differences in nonlinear coupling between emotional valence are mainly located over the left tempo-parietal and pre-frontal cortices for EEG oscillations in the δ,θ,α bands functionally coupled with HRV-LF power. No significant differences between pleasant and unpleasant videos were found for EEG-β,γ and HRV-LF power. Preferred brain areas for the functional nonlinear BHI through HRV-HF power seem to be over the right hemisphere, especially for the δ,α,β oscillations. Also, BHI through EEG-α oscillations seems to mainly occur over the pre-frontal and frontal regions.

## 3. Discussion

We investigated brain–heart interplay (BHI) in healthy young adults and, more specifically, focused on the difference between pleasant and unpleasant high-arousing video through EEG and HRV series. We considered five canonical EEG bands, namely, δ,θ,α,β,γ, and LF and HF powers from HRV series to derive time-varying estimates of synchronous brain and heart activity.

We built on our previous study [35] in which functional linear or nonlinear BHI were investigated by computing MIC, and healthy subjects’ emotions were elicited through images at different valence/arousal levels. Here, we used a more realistic and effective elicitation based on high-arousing videos, and further quantified fully linear or nonlinear functional couplings by combining the Pearson correlation coefficient with MIC, as suggested in [39]. Note that in this study, the SPWVD method was used for the time-varying derivation of HRV-LF and HRV-HF powers. A functional linear BHI refers to a proportional variation between the neural activity of a specific cortical region and in the cardiovascular parasympathetic and/or sympathetic activity. On the other hand, a nonlinear coupling function associates the dynamics of the two systems with a general function, where the input–output proportional variation may not be observed. It is widely accepted that functional BHI may hardly be considered as linear given the multiple feedbacks occurring at many biological and system levels, so knowledge on the actual linear or nonlinear nature of BHI may guide and inform future research on multisystem physiological modelling.

Experimental results are expressed as *p*-value topographic maps, as well as continuous Z-score topographic maps to comply with latest recommendations on *p*-value interpretation and thresholding [45,46]. Results show that functional BHI increases during videos with respect to a resting state considering EEG oscillations up to the β band (<30 Hz). This is in line with previously reported differences in functional BHI over EEG γ oscillations with respect to lower frequencies [28]. In fact, according to a MIC analysis, functional BHI increases in some brain regions with respect to a resting condition following a strong sympathetic elicitation (cold-pressor test) considering EEG oscillations <30 Hz [28]. For the same EEG frequency, MIC values did not show significant differences between positive and negative images for cardiovascular oscillations in the HRV-LF band, whereas differences in the occipital region were found for the HRV-HF band [35]. We speculate that these differences could be due to the use of different elicitation media (images vs. video), or to the different valence/arousal levels implemented in the two experimental setups. Note that EEG γ oscillations may become significant markers of functional BHI when considering a directional brain–heart modelling [9,28] or cortical dynamics exclusively [47].

Consistent with previous studies [35,36,37,38], we found that major BHI differences in processing positive and negative emotional stimuli are associated with EEG oscillations in the θ band. Our findings also suggest that the functional nature of such a BHI is mainly linear, especially occurring over the left-temporal and parietal cortices.

Although it has been pointed out that lateralisation of brain regions could be part of differential positive vs. negative emotional processing [9,48], we did not find major differences in valence between left and right hemispheres for MIC or its derived quantifiers. However, we may speculate that major differences in functional linear BHI seem to occur in the θ band through the left hemisphere, whereas nonlinear BHI tends to occur over the right hemisphere through δ,α and β oscillations. Note that the significant role in BHI of EEG-δ and β oscillations has recently been reported [32].

Finally, at a qualitative level, we report that no major differences in MIC and related quantifiers were found between HRV-LF or HRV-HF powers. This could be due to the fact that both powers seem mainly determined by the parasympathetic system [49], therefore masking possible differential BHI dynamics when considering a non-directional brain–heart modelling.

## 4. Conclusions

In conclusion, functional BHI increases during video emotional processing with respect to a resting state considering EEG oscillations below 30 Hz. No significant changes in functional linear or nonlinear BHI occur between positive and negative videos for EEG oscillations in the γ band. Moreover, high-arousing positive videos increase BHI through a functional linear coupling with EEG oscillations in the θ band, especially over the left-temporal and parietal cortices. Differential functional nonlinear coupling between positive and negative valence seems to mainly occur with EEG oscillations in the δ,θ,α bands through sympatho-vagal (HRV-LF) dynamics, whereas preferred brain areas for a functional nonlinear interplay seem to occur over the right hemisphere, especially for the δ,α,β oscillations, through parasympathetic activity (HRV-HF). In addition, the functional interactions between cardiovascular dynamics and brain oscillations in the δ and α bands over the prefrontal region seem to be primarily nonlinear. From a physiological viewpoint, the specific phenomena underlying the different linear and nonlinear brain–heart interplay are still unknown to us. Finally, BHI changes between positive and negative videos through EEG-α oscillations seem to mainly occur over the pre-frontal and frontal regions.

This study provides novel insights into synchronous cortical and heartbeat dynamics during emotional elicitation, also suggesting that a nonlinear analysis is needed to fully characterize functional BHI.

## 5. Materials and Methods

### 5.1. Experimental Setup

Thirty healthy subjects (15 females; 26.3 years on average) signed an informed consent and volunteered to participate in the study. Volunteers sat comfortably on a chair for a few minutes also to make them reach a hemodynamic stabilization. Then, sensors for a one-lead ECG and a 128-channel EEG recordings were placed onto the subjects. The signals sampling frequency was set at 500 Hz.

For each subject, a video projector was used to perform an emotional elicitation using video clips. The experimental protocol started with a 3-min resting state session with closed eyes, followed by a 4:30-min of emotional video elicitation. This eliciting session comprised the following sub-sessions: (i) a 1:30-min session with high-arousing and unpleasant videos, (ii) a 1:30-min session with high-arousing and pleasant videos, and (iii) a 1:30-min session with neutral videos. The subjects were presented with arousing elicitation sessions in a randomized order. Videos were selected from a set that was presented to a group of 20 healthy subjects. Each subject was then asked to score the videos in terms of arousal and valence level, and only videos with highest arousal and highest positive/negative valence were chosen for further use. None of the subjects participating to this preliminary assessment was enrolled for the subsequent physiological data recordings. All experimental procedures were approved by the local ethical committee Area Vasta Nord-Ovest Toscana.

### 5.2. ECG and EEG Data Pre-Processing

ECG processing steps to derive HRV series are detailed in [35]. Briefly, R-peaks were identified using the well-known Pan-Tompkins algorithm, and algorithmic and physiological artefacts from each RR interval series were corrected using a procedure based on a local log-likelihood point-process statistics [50].

For the EEG pre-processing, a subset of 90 EEG channels was selected to improve the subsequent Independent Component Analysis (ICA) performance while avoiding so-called over-learning issues [51]. A detailed map of the 90 EEG channels selected for further analyses is reported in the Supplementary Information. We exploited a pipeline structure called HAPPE that is thoroughly described in [52]. Briefly, bad channels rejection is implemented by calculating the normed joint probability of the mean log power from 1 to 125 Hz, and EEG channels belonging to the external 1% tails of the distribution are not considered for further analyses. Bad channels are recovered through a spherical interpolation algorithm using neighbour EEG data. EEG signals are then high-pass filtered at 1 Hz, and electrical noise at 50 Hz and possibly at 100 Hz is rejected through a multi-taper regression approach [52]. Afterwards, wavelet-enhanced ICA steps are performed to correct for EEG artefacts, including eye- or muscle-related activities and discontinuities in the recording. This correction is further refined using a machine learning-based approach applied to ICA components [52]. A re-reference of all EEG data to the average calculated from all of the channels (i.e., average re-reference) was the final processing step.

### 5.3. EEG and HRV Time-Varying Spectra

A time course estimation of the power spectrum was performed through short-time Fourier transform, using a Hanning window of 1 s and 1 s step size on EEG series, whereas a smoothed pseudo-Wigner-Ville distribution (SPWVD) method was applied to HRV series [53]. The latter choice is justified by the time-frequency resolution of SPWVD, as well as by the low variance of the estimated power spectra.

### 5.4. Maximum Information Coefficient (MIC) and Linear–Nonlinear Coupling

MIC [39] quantifies a linear or nonlinear coupling between two series, and ranges within [0,1].

Starting from the scatterplot of the ordered pairs of two vectors *x* and *y*, a number of rows and columns can be drawn to obtain different partitions and define the following metrics:

(1) $$m_{x\times y}=\frac{\underset{g\in G_{x\times y}}{\max\left\{I_{g}\right\}}}{\log\min\{x,y\}}$$

with x≤n and y≤n, where *n* is the dimension of the vectors, $G_{x\times y}$ the sample of all the possible partitions with *x* rows and *y* columns, and $I_{g}$ the mutual information of a specific partition.

MIC is then calculated as the maximum $m_{x\times y}$ over all the ordered pairs (x,y). Formally, it is possible to estimate $MIC=\max_{xy<B}\left\{m_{x\times y}\right\}$ with *B* empirically defined as $B=n^{0.6}$ [39].

It is then possible to derive metrics of nonlinear coupling exclusively as: MIC$-\rho^{2}$ by estimating the well-known Pearson linear correlation coefficient ρ.

### 5.5. Statistical Analysis

Variable statistical comparison between positive and negative elicitation sessions was performed through non-parametric Wilcoxon tests for paired samples, with a null hypothesis of equal medians between sessions. Results from this analysis are shown as topographical maps where green areas indicate non-significant *p*-value (p>0.05). Brain areas with p<0.05 are indicated with a different colour depending on the associated Z statistics from the Wilcoxon test. All *p*-values were corrected for a multiple comparison using the permutation test, with a total of 1000 permutations.

## Figures and Tables

**Table 1:** Descriptive EEG and heartbeat dynamics and HRV parameter statistics.

	Power	Units	Resting Phase	Positive Valence	Negative Valence
EEG	δ	$\left(mV^{2}\right)$	1.608±1.714	1.000±1.044	1.045±1.201
	θ	$\left(mV^{2}\right)$	0.4834±0.5515	0.2989±0.3139	0.2964±0.3125
	α	$\left(mV^{2}\right)$	1.414±1.727	0.405±0.3359	0.400±0.3233
	β	$\left(mV^{2}\right)$	0.3395±0.3895	0.213±0.1821	0.210±0.173
	γ	$\left(mV^{2}\right)$	0.475±1.264	0.332±0.9024	0.526±1.659
HRV	HF	$\left(mV^{2}\right)$	1.432±0.1455	0.9907±0.0418	1.241±0.0459
	LF	$\left(mV^{2}\right)$	1.375±0.2253	0.8439±0.0528	1.197±0.0617
	HR	(sec)	0.8276±0.066	0.8356±0.0526	0.8421±0.0637

values are expressed as mean ± std.

## Supplementary Materials

The following are available at https://www.mdpi.com/1099-4300/21/9/892/s1, Figure S1: Topographic maps of Z-score values from the Wilcoxon non-parametric test for paired data applied to MIC estimates between resting state and positive video elicitations, Figure S2: Topographic maps of Z-score values from the Wilcoxon non-parametric test for paired data applied to MIC estimates between resting state and negative video elicitations, Figure S3: MIC values topographic maps calculated over a 1.5 min high-arousing, positive video elicitation (median among all subjects for each EEG channel). The top panels refer to the functional interaction between the HRV-HF power and EEG oscillations at all bands, whereas the bottom panels refer to the HRV-LF power, Figure S4: MIC values topographic maps calculated over a 1.5 min high-arousing, negative video elicitation (median among all subjects for each EEG channel). The top panels refer to the functional interaction between the HRV-HF power and EEG oscillations at all bands, whereas the bottom panels refer to the HRV-LF power, Figure S5: Topographic distribution of the relative variation between MIC values extracted during positive and negative elicitation phases, Figure S6: HydroCel Geodesic Sensor Net. External EEG channels in the red circle were discarded for further coupling analyses, Table S1: EEG channels associated with statistically significant brain-heart interplay changes between positive and negative elicitation sessions, grouped by HRV and EEG frequency bands. EEG channel labels and numbers refer to the EGI channel map shown below.
