# Supplementary material for: Functional Linear and Nonlinear Brain–Heart Interplay during Emotional Video Elicitation: A Maximum Information Coefficient Study
Source: Entropy (Basel). 2019 Sep 14;21(9):892. doi: 10.3390/e21090892 (PMC7515428; doi:10.3390/e21090892)
Supplement: Supplementary file 1 [file entropy-21-00892-s001.zip › entropy-556242-supplementary.pdf]

# Supplementary Materials: Functional Linear and Nonlinear Brain–Heart Interplay during Emotional Video Elicitation: A Maximum Information Coefficient Study

**Vincenzo Catrambone *, Alberto Greco, Enzo Pasquale Scilingo and Gaetano Valenza**

## 1. MIC Comparison between Resting State and Emotional Videos

Below are the results from the statistical comparison on MIC values between resting state and emotional video using Wilcoxon non-parametric test for paired data.

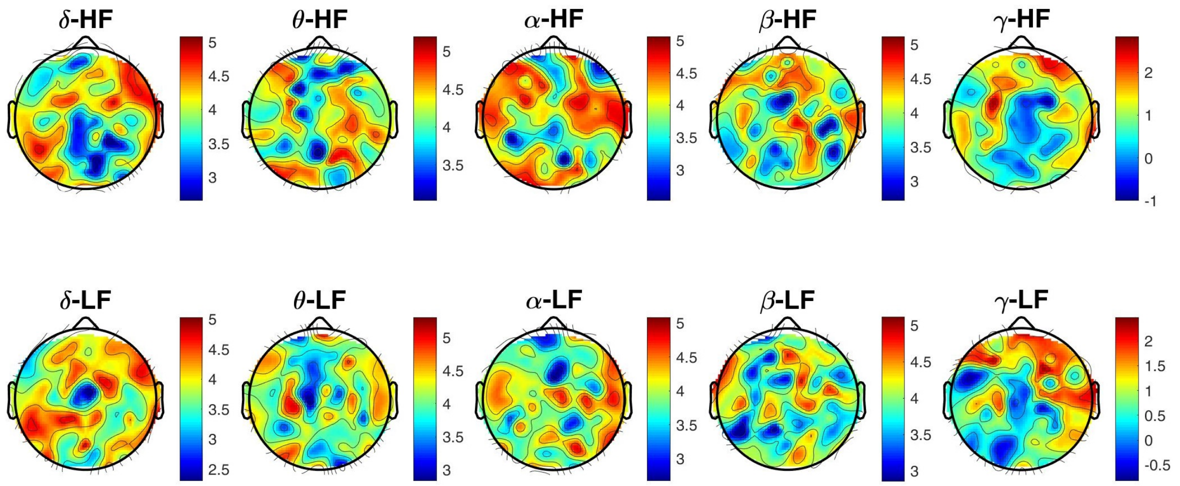


**Figure S1.** Topographic maps of Z-score values from the Wilcoxon non-parametric test for paired data applied to MIC estimates between resting state and positive video elicitations.

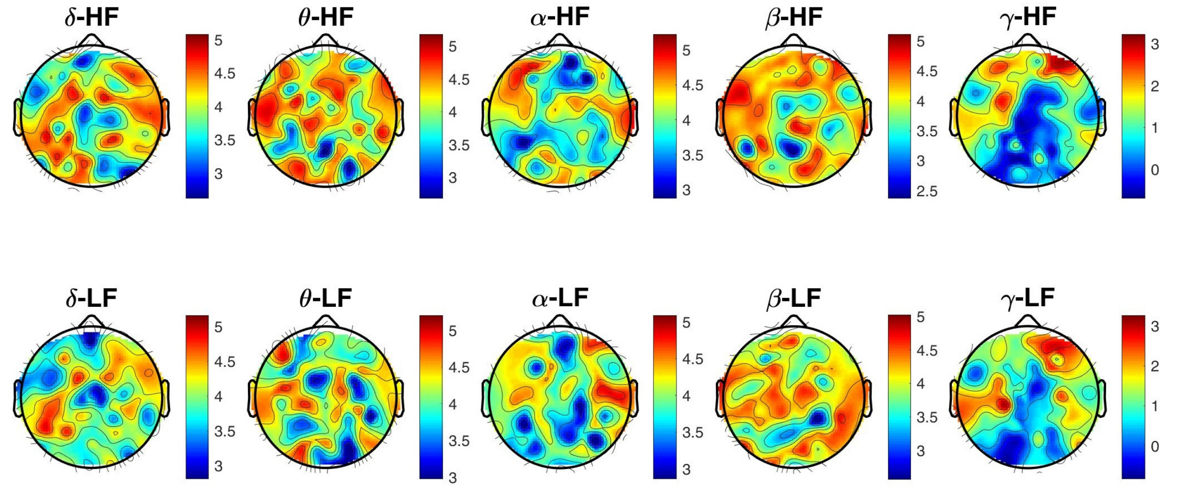


**Figure S2.** Topographic maps of Z-score values from the Wilcoxon non-parametric test for paired data applied to MIC estimates between resting state and negative video elicitations.

## 2. Group-Wise MIC Values during Emotional Videos

Below are the group-wise (median) MIC values for positive and negative videos.

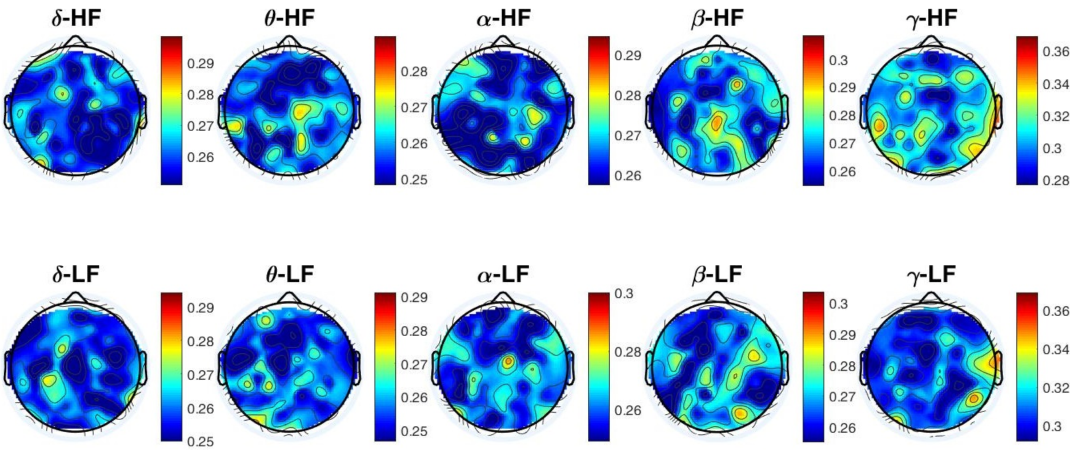


**Figure S3.** MIC values topographic maps calculated over a 1.5 min high-arousing, positive video elicitation (median among all subjects for each EEG channel). The top panels refer to the functional interaction between the HRV-HF power and EEG oscillations at all bands, whereas the bottom panels refer to the HRV-LF power.

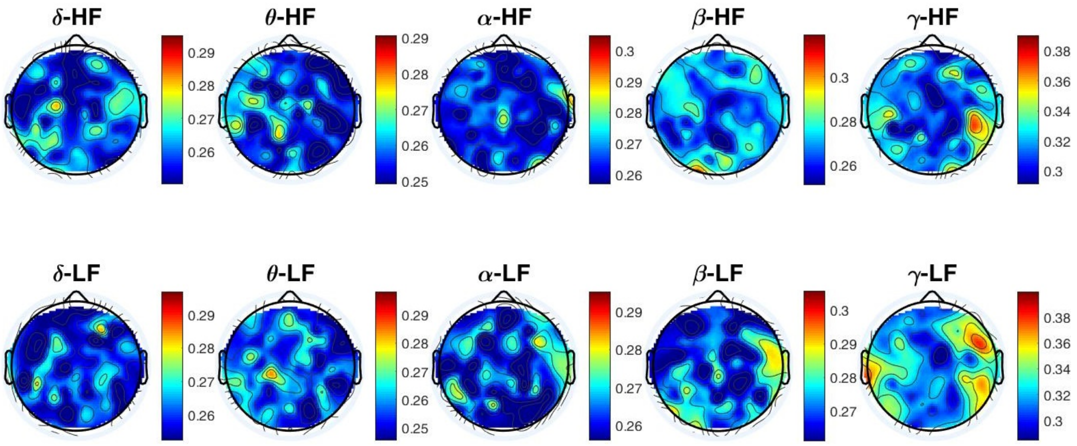


**Figure S4.** MIC values topographic maps calculated over a 1.5 min high-arousing, negative video elicitation (median among all subjects for each EEG channel). The top panels refer to the functional interaction between the HRV-HF power and EEG oscillations at all bands, whereas the bottom panels refer to the HRV-LF power.

## 3. Quantitative Analysis of Changes between Sessions

The following figure shows the topographic distribution of MIC relative variation between positive and negative emotional video elicitation normalized by the average between MIC values extracted during the two stimulation phases. The relative variation (RV) has been calculated as follows:

$$RV = \frac{MIC_p - MIC_n}{\frac{MIC_p + MIC_n}{2}}$$

where $MIC_p$ and $MIC_n$ stand respectively for the MIC extracted during positive and negative video elicitations.

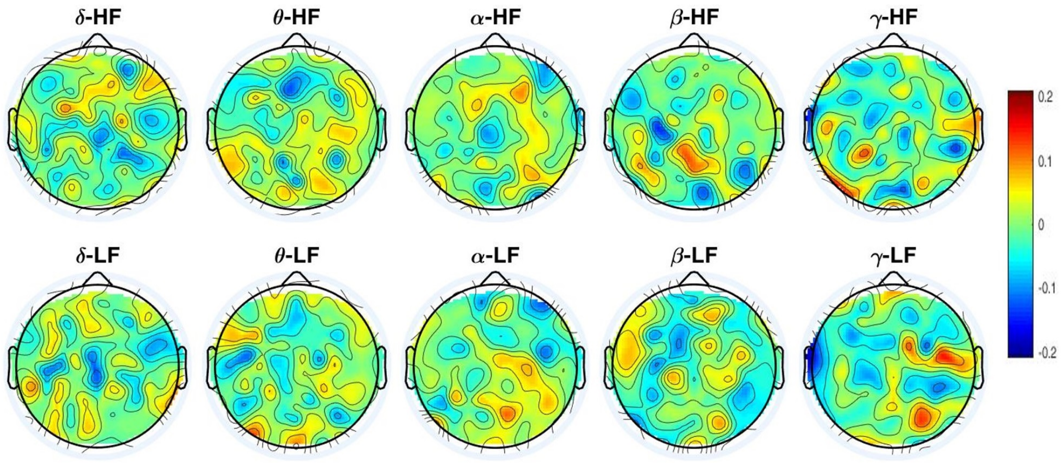


**Figure S5.** Topographic distribution of the relative variation between MIC values extracted during positive and negative elicitation phases.

In terms of location of the electrodes found significant in the statistical analysis, it is hereby provided a table listing EEG channels showing significant changes, grouped by EEG and HRV frequency bands.

**Table S1.** EEG channels associated with statistically significant brain-heart interplay changes between positive and negative elicitation sessions, grouped by HRV and EEG frequency bands. EEG channel labels and numbers refer to the EGI channel map shown below.

| | **HRV-LF** | **HRV-HF** |
|---|---|---|
| EEG-$\delta$ | [C3; 41; 93; T4; 116] | [3; 13; 41; 47; 86; T4; 109] |
| EEG-$\theta$ | [31; 35; 65; 75; 91] | [12; 71; 84; 87; C4] |
| EEG-$\alpha$ | [2; 18; T5; 97] | [31; 85; 115] |
| EEG-$\beta$ | [13; 50; T5; 102; 116] | [Pz; 97] |
| EEG-$\gamma$ | No significant channels | [65] |

## 4. EGI Channel Map

Below is the channel map of the EGI net system that was used in this study.

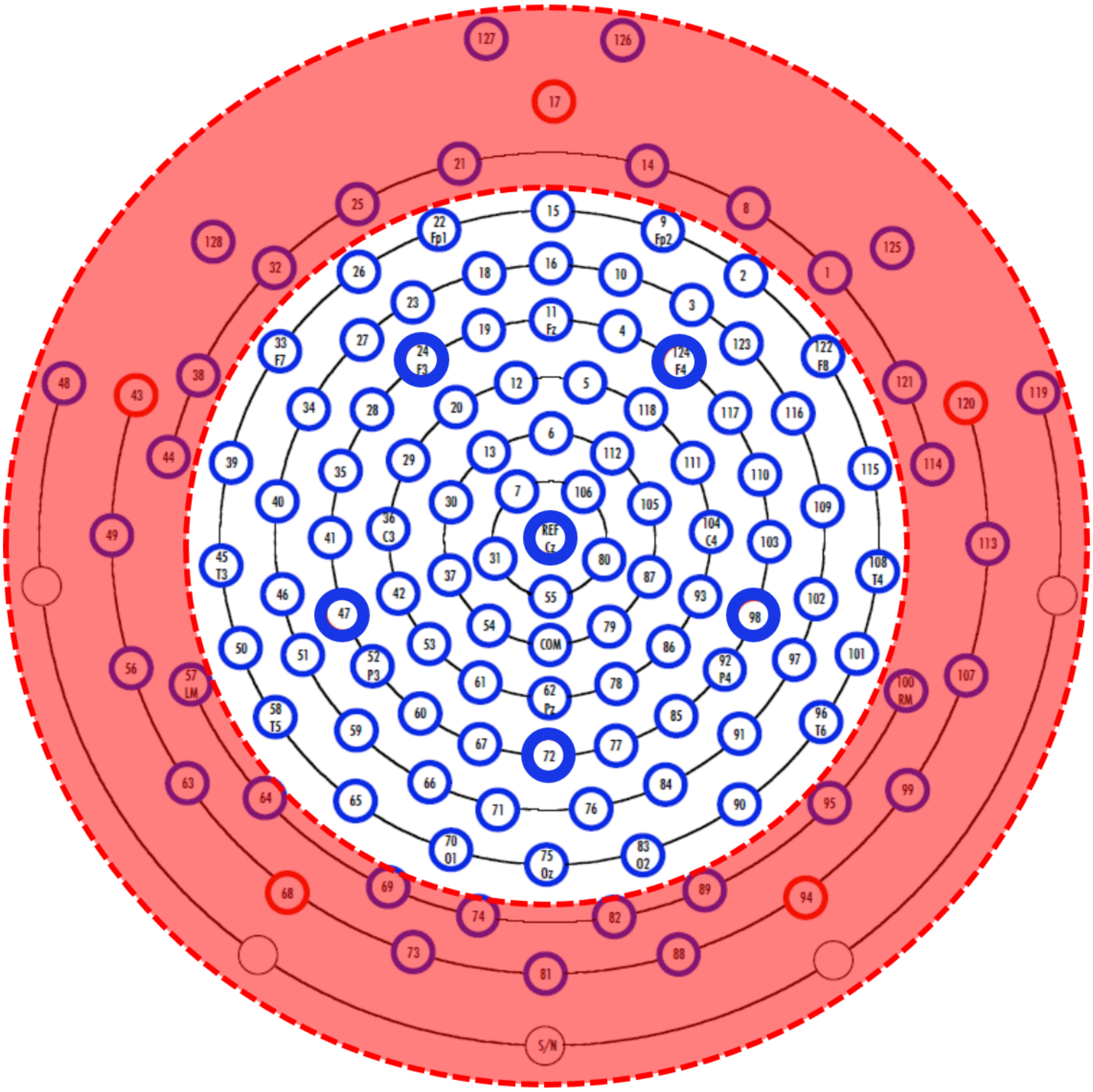


**Figure S6.** HydroCel Geodesic Sensor Net. External EEG channels in the red circle were discarded for further coupling analyses.
