# Supplementary material for: Functional Linear and Nonlinear Brain–Heart Interplay during Emotional Video Elicitation: A Maximum Information Coefficient Study
Source: Entropy (Basel). 2019 Sep 14;21(9):892. doi: 10.3390/e21090892 (PMC7515428; doi:10.3390/e21090892)
Supplement: Supplementary file 1 [file entropy-21-00892-s001.zip › figures/NonLin_pvs_PN_perm-eps-converted-to.pdf]

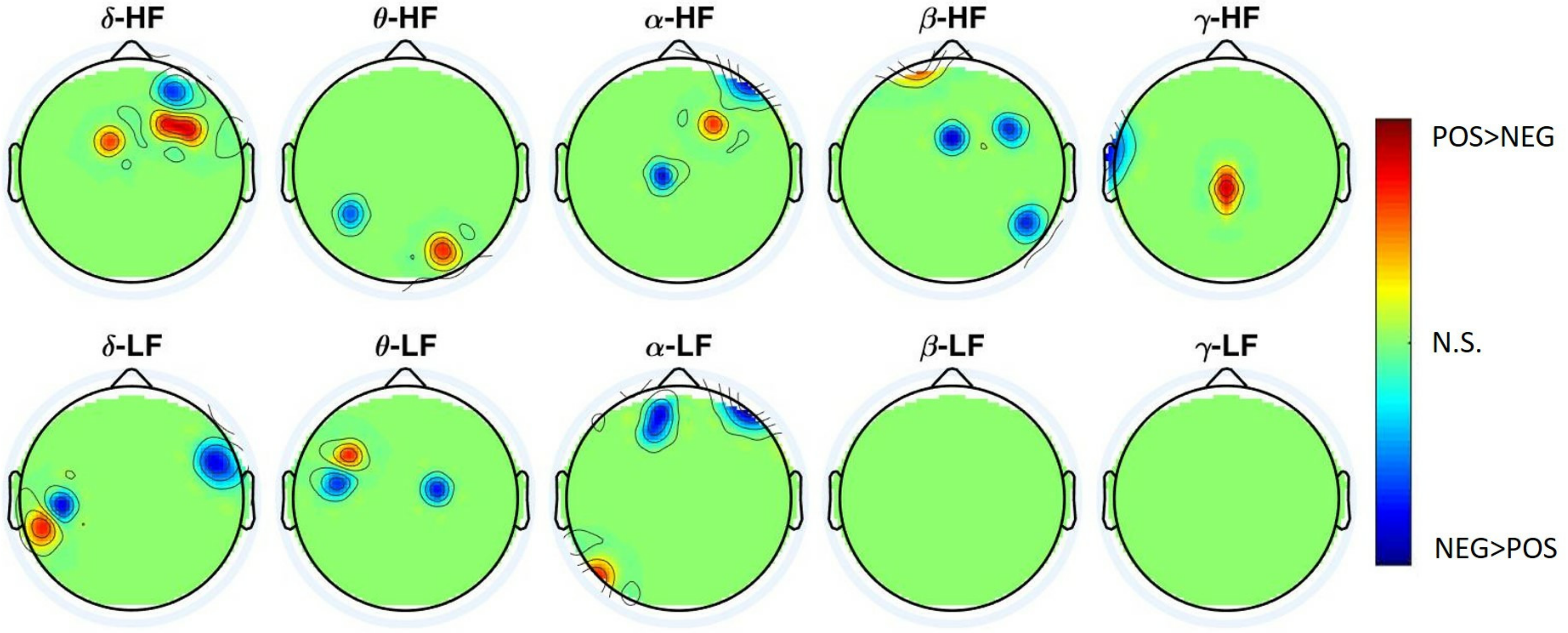
δ-HF
θ-HF
α-HF
β-HF
γ-HF
δ-LF
θ-LF
α-LF
β-LF
γ-LF
POS>NEG
N.S.
NEG>POS
