# Supplementary figures and images for: Functional Linear and Nonlinear Brain–Heart Interplay during Emotional Video Elicitation: A Maximum Information Coefficient Study
Source: Entropy (Basel). 2019 Sep 14;21(9):892. doi: 10.3390/e21090892 (PMC7515428; doi:10.3390/e21090892)

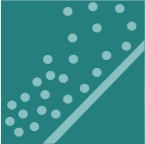

*entropy*

Supplement: Supplementary file 1 [file entropy-21-00892-s001.zip › Definitions/entropy-logo-eps-converted-to.pdf]

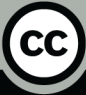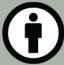

BY

Supplement: Supplementary file 1 [file entropy-21-00892-s001.zip › Definitions/logo-ccby-eps-converted-to.pdf]

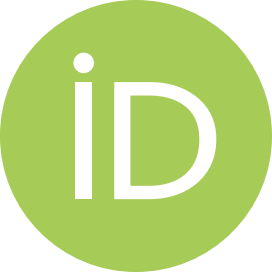

Supplement: Supplementary file 1 [file entropy-21-00892-s001.zip › Definitions/logo-orcid-eps-converted-to.pdf]

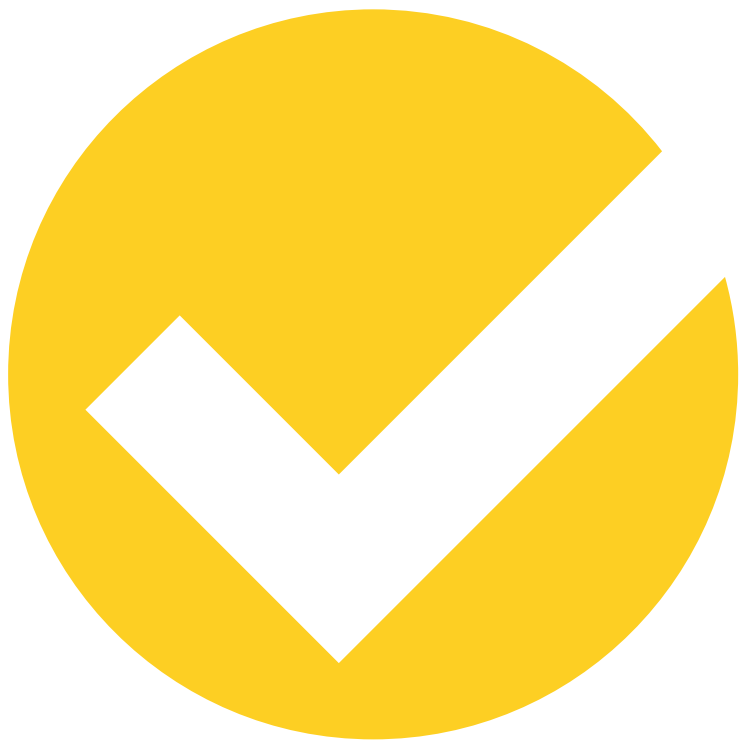

check for  
updates

Supplement: Supplementary file 1 [file entropy-21-00892-s001.zip › Definitions/logo-updates.pdf]

$\delta$ -HF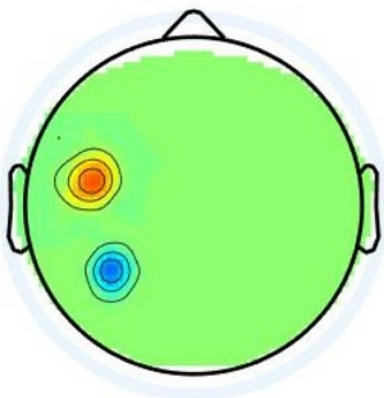 $\theta$ -HF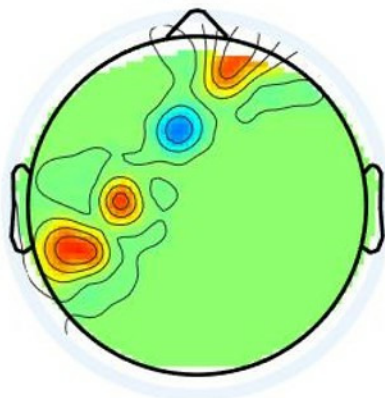 $\alpha$ -HF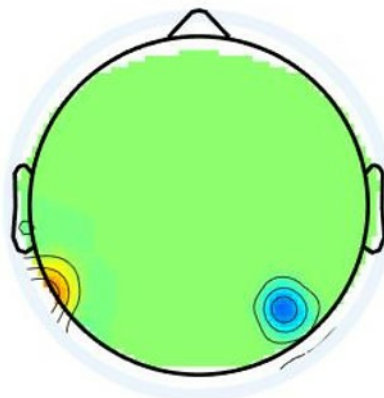 $\beta$ -HF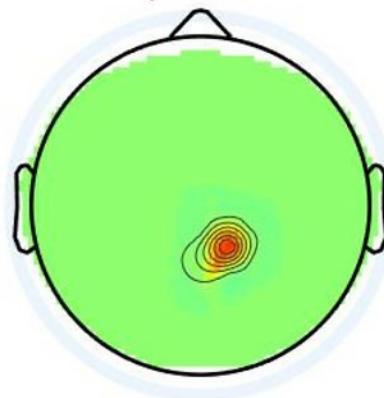 $\gamma$ -HF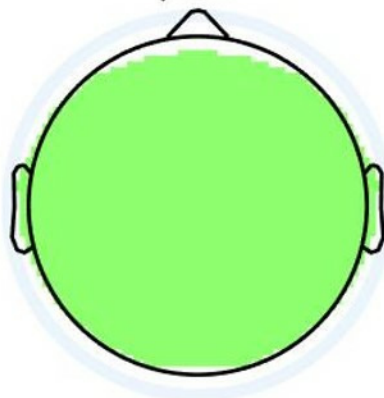 $\delta$ -LF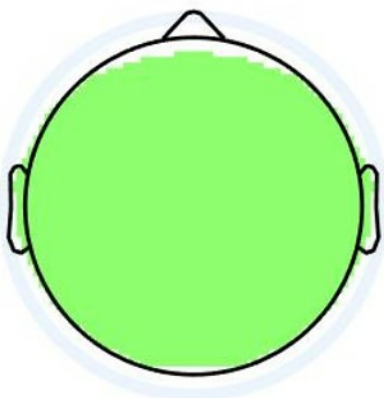 $\theta$ -LF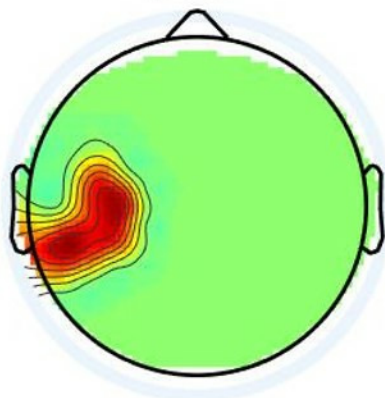 $\alpha$ -LF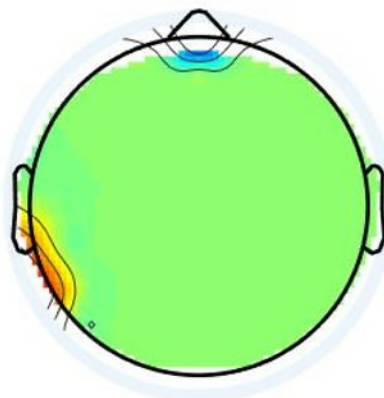 $\beta$ -LF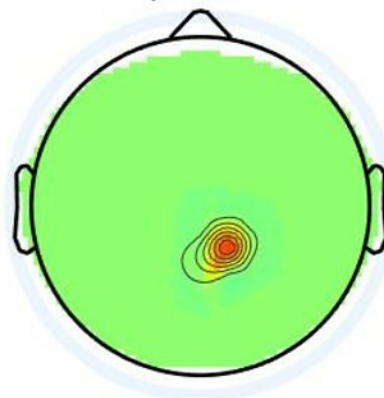 $\gamma$ -LF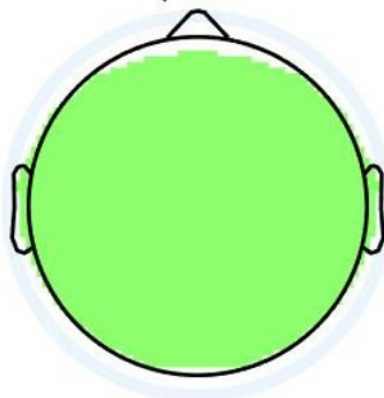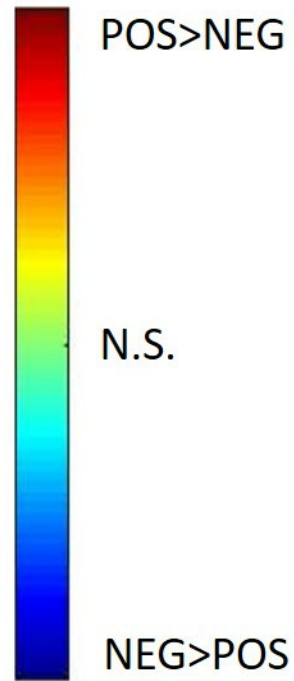

Supplement: Supplementary file 1 [file entropy-21-00892-s001.zip › figures/Lin_pvs_PN_perm-eps-converted-to.pdf]

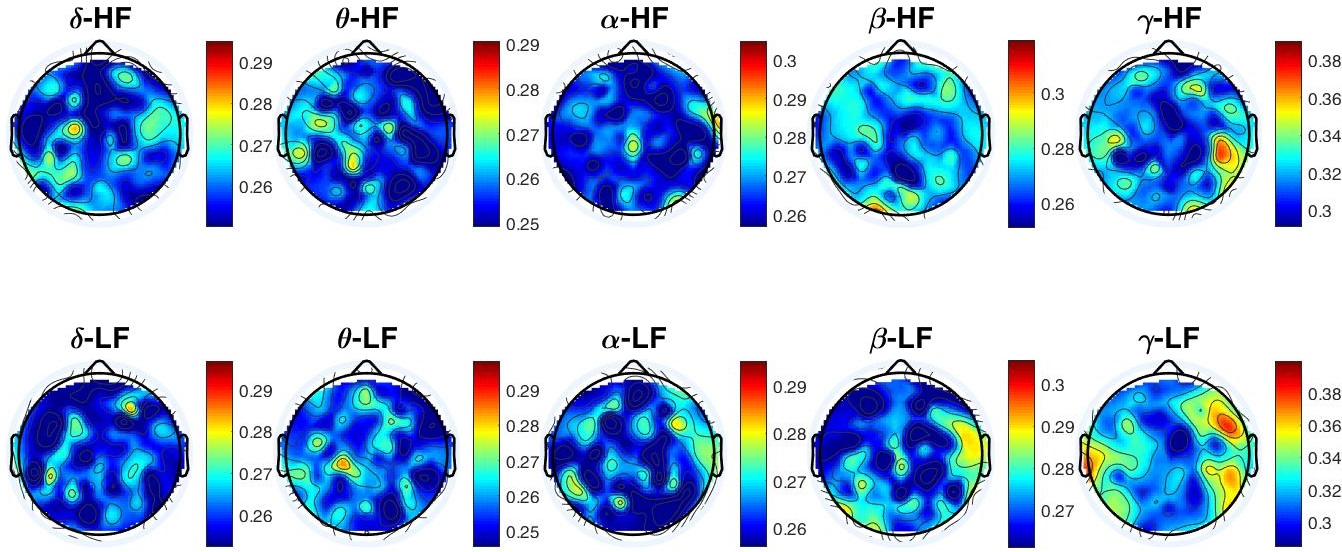

Supplement: Supplementary file 1 [file entropy-21-00892-s001.zip › figures/MIC_neg.jpg]

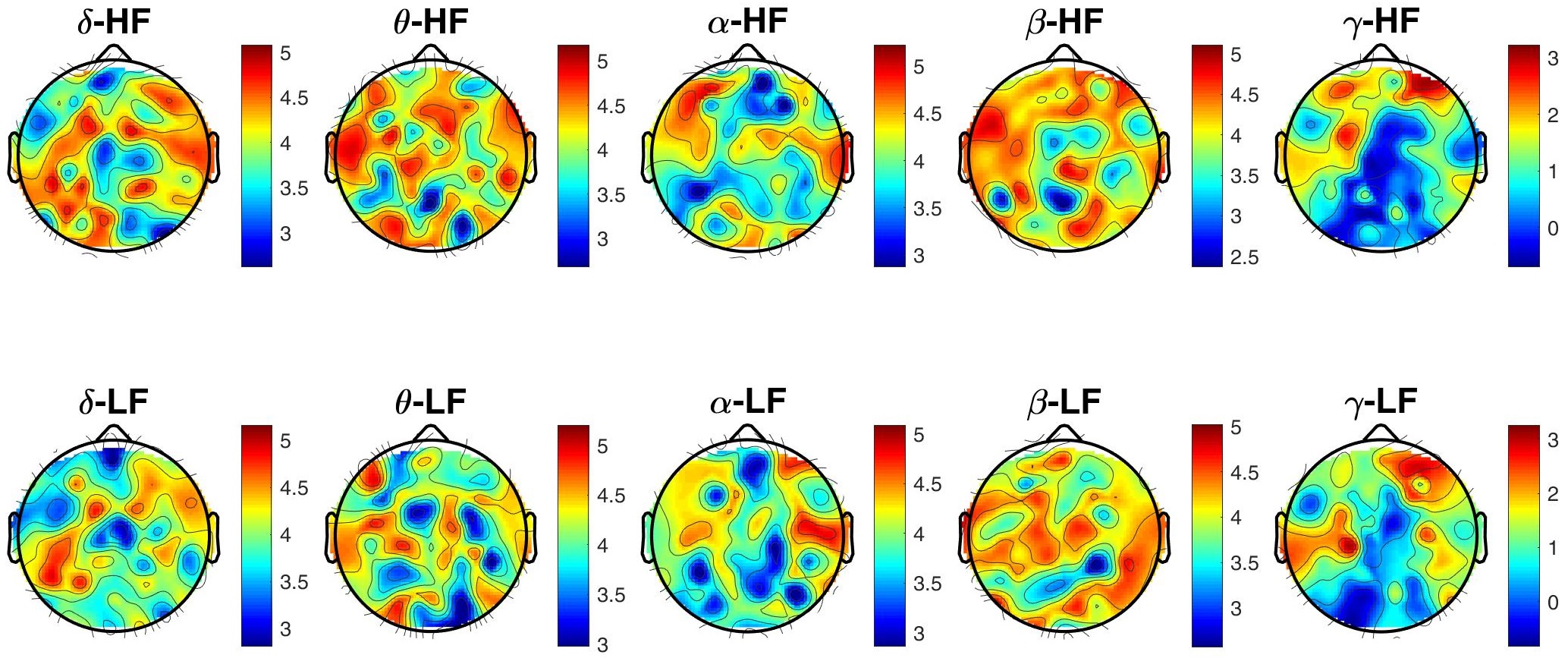

Supplement: Supplementary file 1 [file entropy-21-00892-s001.zip › figures/MIC_NegVsRest.jpg]

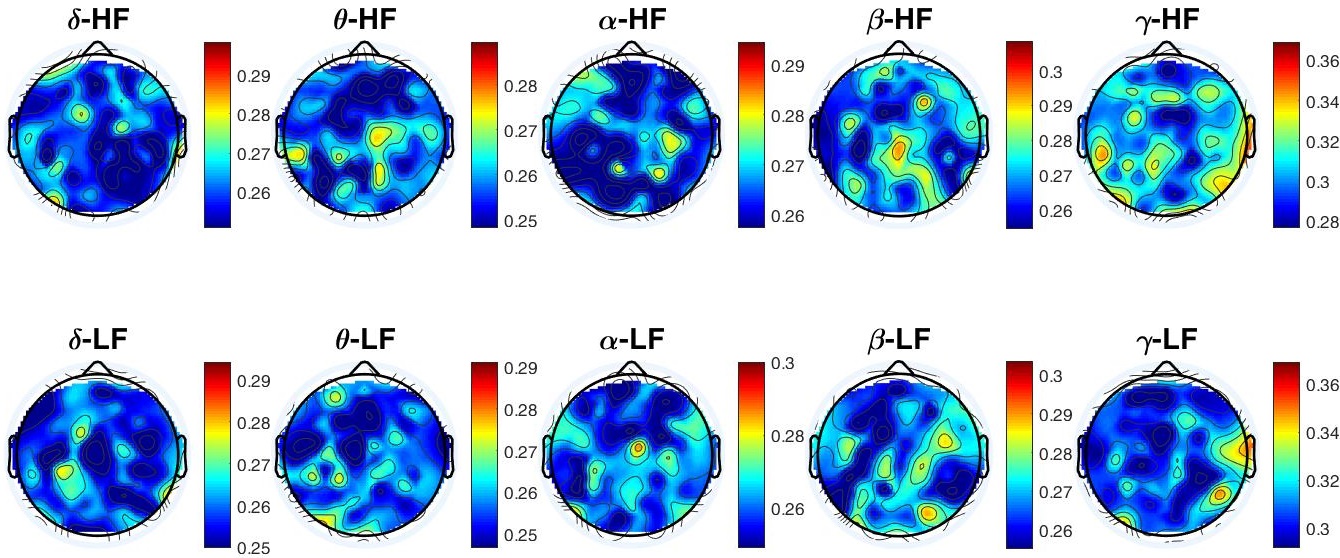

Supplement: Supplementary file 1 [file entropy-21-00892-s001.zip › figures/MIC_pos.jpg]

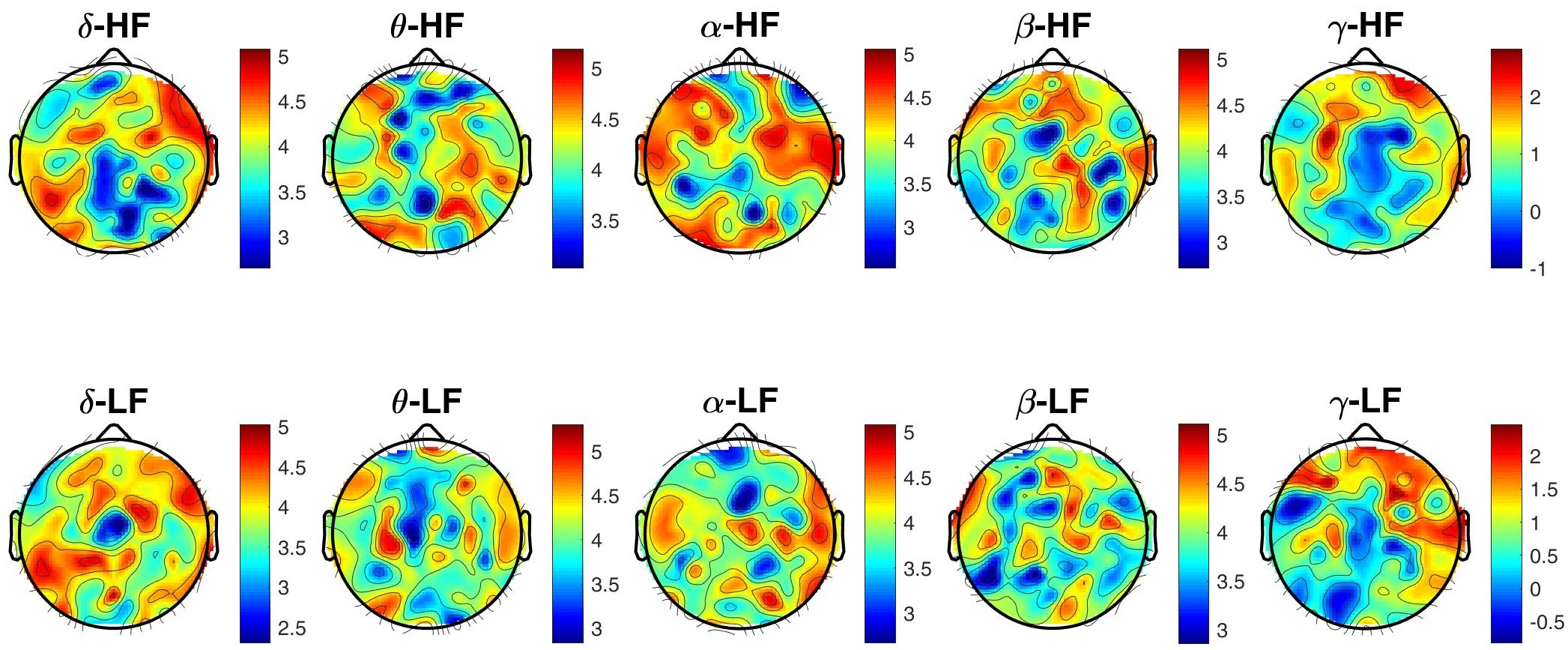

Supplement: Supplementary file 1 [file entropy-21-00892-s001.zip › figures/MIC_PosVsRest.jpg]

$\delta$ -HF $\theta$ -HF $\alpha$ -HF $\beta$ -HF $\gamma$ -HF $\delta$ -LF $\theta$ -LF $\alpha$ -LF $\beta$ -LF $\gamma$ -LF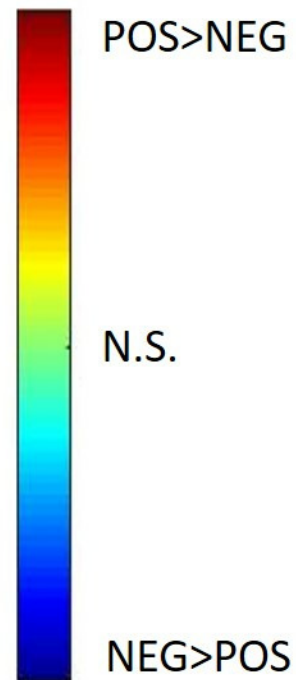

Supplement: Supplementary file 1 [file entropy-21-00892-s001.zip › figures/MIC_pvs_PN_perm-eps-converted-to.pdf]

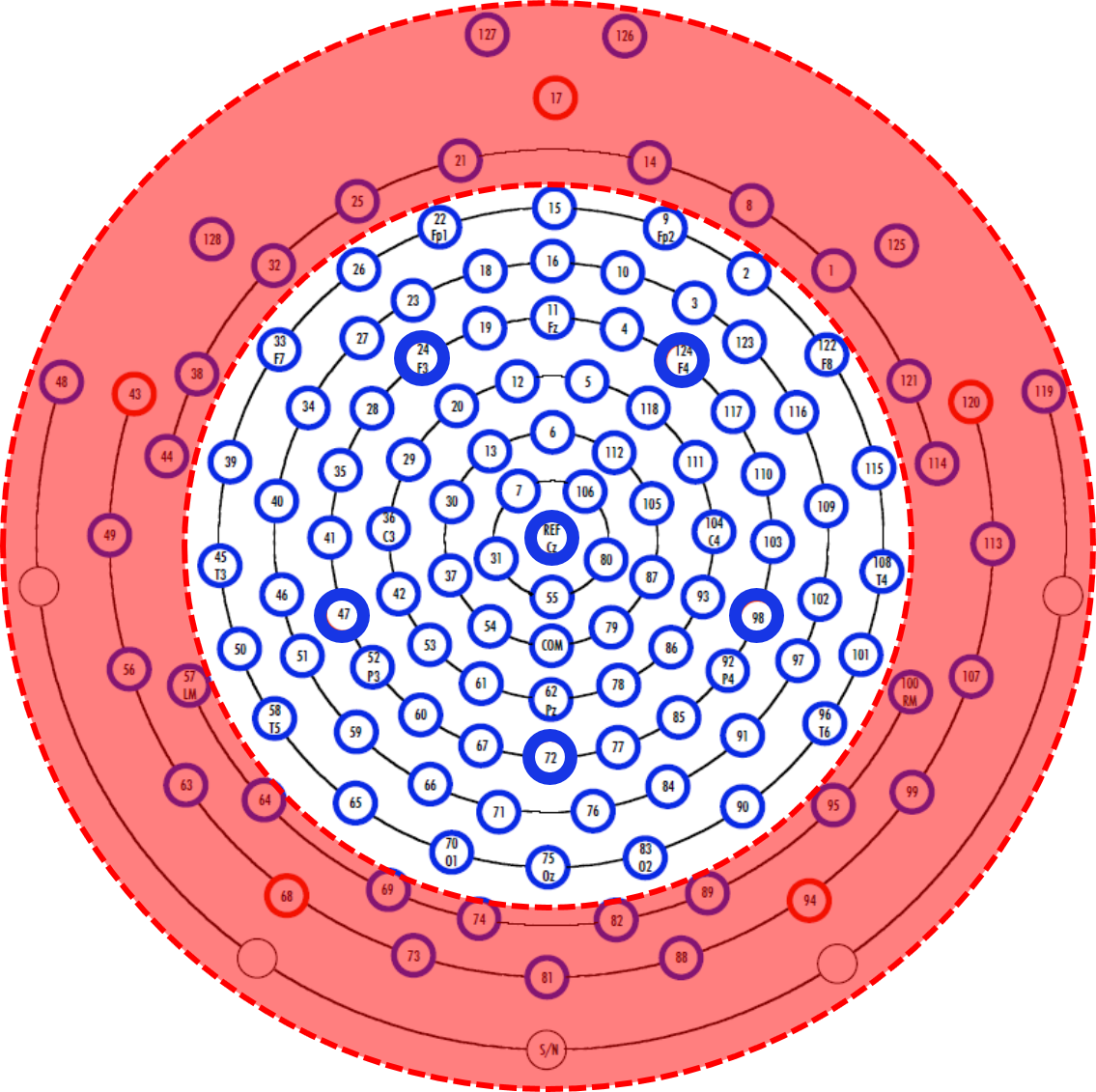

Supplement: Supplementary file 1 [file entropy-21-00892-s001.zip › figures/MyChannelMap.png]

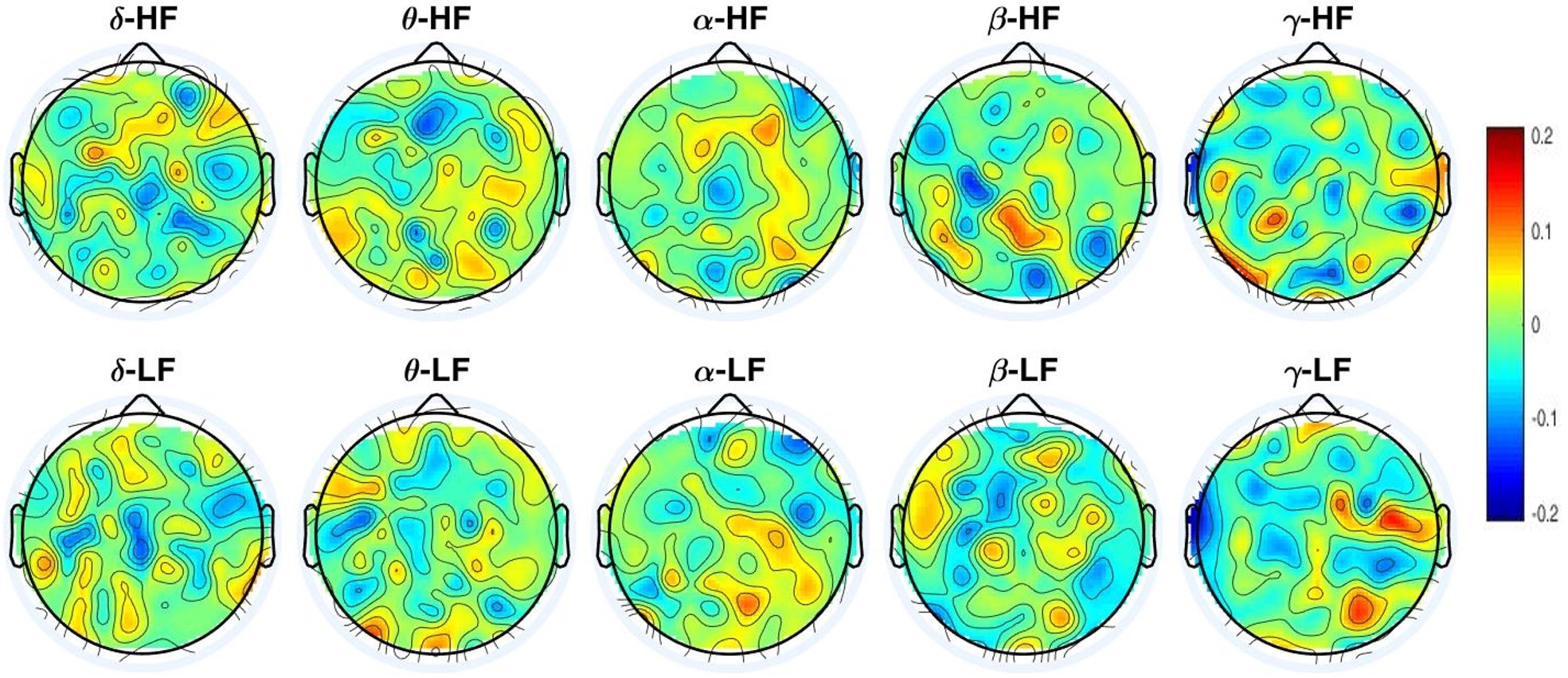

Supplement: Supplementary file 1 [file entropy-21-00892-s001.zip › figures/relative_variation_MIC.jpg]

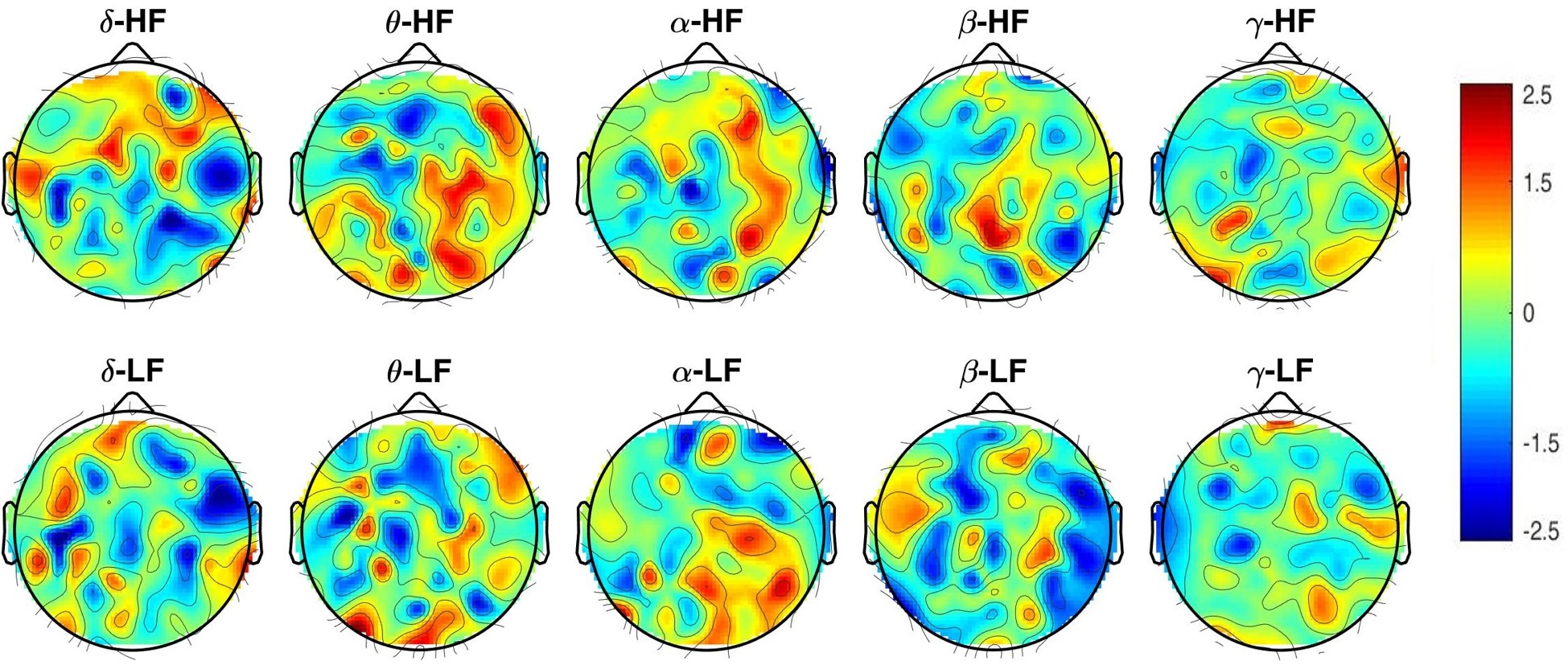

Supplement: Supplementary file 1 [file entropy-21-00892-s001.zip › figures/Zvalues_MIC.jpg]

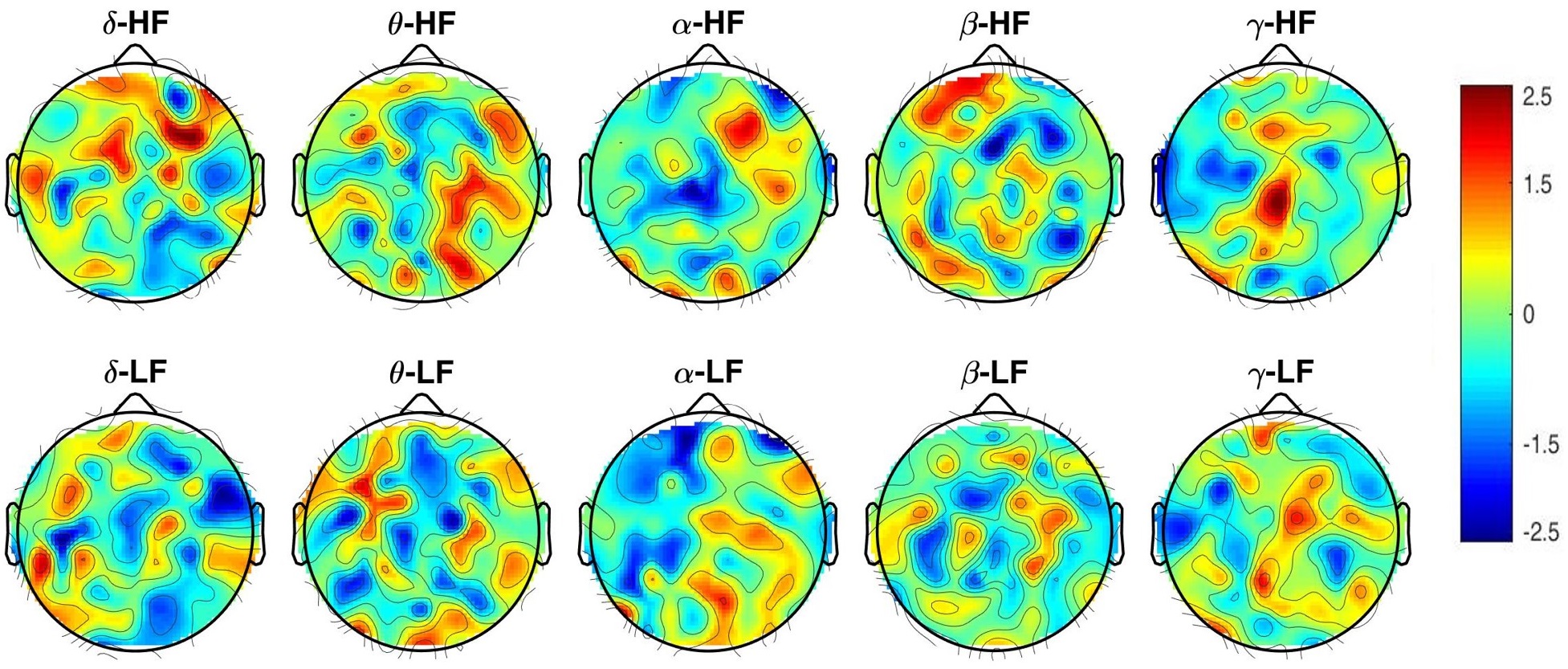

Supplement: Supplementary file 1 [file entropy-21-00892-s001.zip › figures/Zvalues_NonLinearity.jpg]

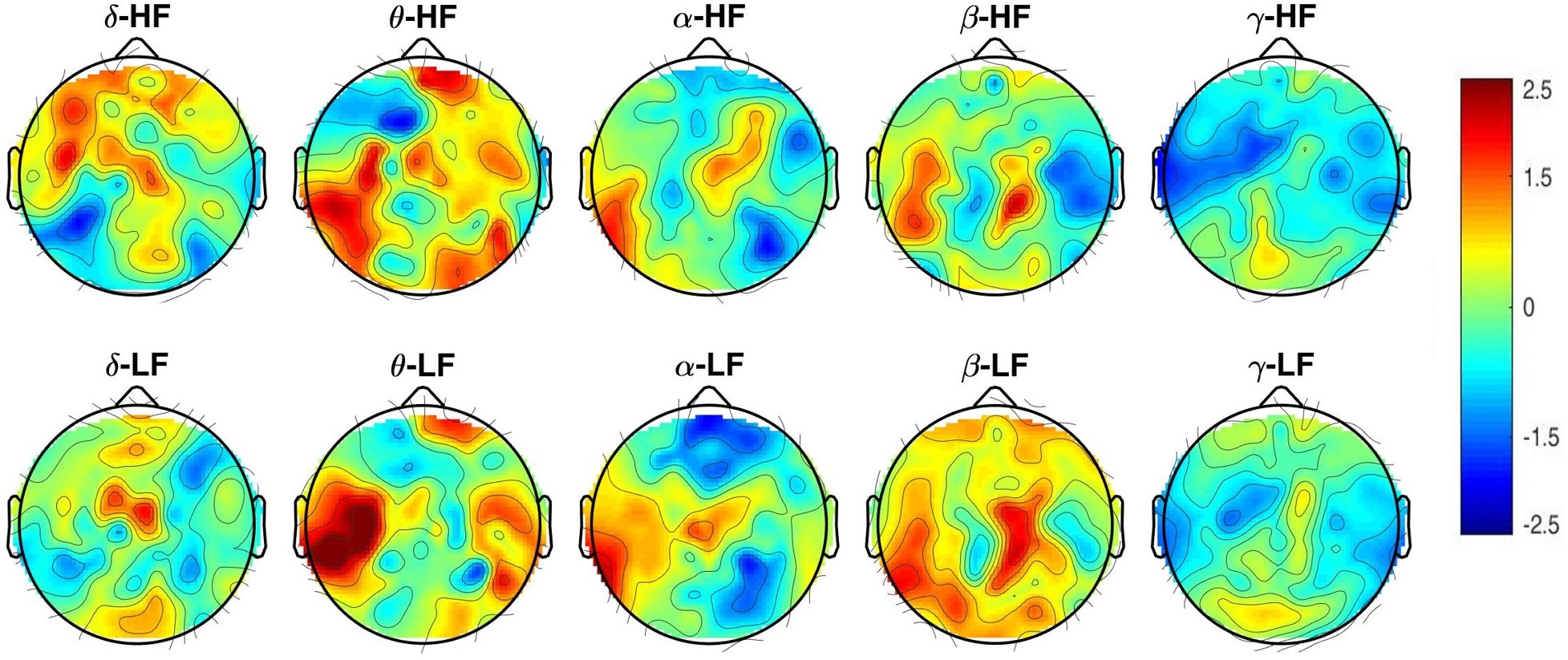

Supplement: Supplementary file 1 [file entropy-21-00892-s001.zip › figures/Zvalues_Rho.jpg]
